# Preoperative prediction of pancreatic neuroendocrine tumor grade based on ^68^Ga-DOTATATE PET/CT

**DOI:** 10.1007/s12020-023-03515-3

**Published:** 2023-09-16

**Authors:** Jiao Ma, Xiaoyong Wang, Mingsong Tang, Chunyin Zhang

**Affiliations:** 1 Department of Nuclear Medicine, The Affilliated Hospital of Southwest Medical University, Luzhou, 646000 Sichuan PR China; 2 Department of Radiology, The Affilliated Hospital of Southwest Medical University, Luzhou, 646000 Sichuan PR China; 3 Nuclear Medicine and Molecular Imaging Key Laboratory of Sichuan Province, Luzhou, 646000 Sichuan PR China; 4 Academician (expert) Workstation of Sichuan Province, Luzhou, 646000 Sichuan PR China

**Keywords:** Neoplasm grading, Pancreatic neuroendocrine tumors, Radiomics, ^68^Ga-DOTATATE PET/CT

## Abstract

**Objective:**

To establish a prediction model for preoperatively predicting grade 1 and grade 2/3 tumors in patients with pancreatic neuroendocrine tumors (PNETs) based on ^68^Ga-DOTATATE PET/CT.

**Methods:**

Clinical data of 41 patients with PNETs were included in this study. According to the pathological results, they were divided into grade 1 and grade 2/3. ^68^Ga-DOTATATE PET/CT images were collected within one month before surgery. The clinical risk factors and significant radiological features were filtered, and a clinical predictive model based on these clinical and radiological features was established. 3D slicer was used to extracted 107 radiomic features from the region of interest (ROI) of ^68^Ga-dotata PET/CT images. The Pearson correlation coefficient (PCC), recursive feature elimination (REF) based five-fold cross validation were adopted for the radiomic feature selection, and a radiomic score was computed subsequently. The comprehensive model combining the clinical risk factors and the rad-score was established as well as the nomogram. The performance of above clinical model and comprehensive model were evaluated and compared.

**Results:**

Adjacent organ invasion, N staging, and M staging were the risk factors for PNET grading (*p* < 0.05). 12 optimal radiomic features (3 PET radiomic features, 9 CT radiomic features) were screen out. The clinical predictive model achieved an area under the curve (AUC) of 0.785. The comprehensive model has better predictive performance (AUC = 0.953).

**Conclusion:**

We proposed a comprehensive nomogram model based on ^68^Ga-DOTATATE PET/CT to predict grade 1 and grade 2/3 of PNETs and assist personalized clinical diagnosis and treatment plans for patients with PNETs.

## Introduction

Pancreatic neuroendocrine tumors (PNETs) originate from peptide-secreting neurons and neuroendocrine cells, accounting for 2~5% of all pancreatic tumors [1]. But in recent years, the incidence rate and prevalence of PNETs have shown a significant upward trend [2, 3]. The 2019 WHO classification divided neuroendocrine tumors (NETs) into three grades (grade1, grade2, and grade3) based on the Ki-67 proliferation index and mitotic rate [4]. PNETs of varying histological grades typically indicate different biological invasiveness, which strongly correlates with prognosis [5, 6]. Accurate histological diagnosis and grading are considered to have a great impact on the prognostic assessment and treatment selection of PNETs [7].

However, the histological grades are usually obtained through histopathological exams after surgery. Surgery plays a crucial role in the treatment of PNETs. But when designing a surgical plan, several factors need to be taken into account, including tumor grade and stage, general condition of the patient, clinical symptoms, functional characteristics, and genetic correlations [8, 9]. Some biochemical indicators such as chromogranin A, neuron-specificenolase, and progastrin-releasing peptide can assist in the diagnosis of NETs and evaluate the curative effect and prognosis of some patients, but their diagnostic effects are affected by many factors, and the overall accuracy and sensitivity are not high [7, 10, 11]. Endoscopic ultrasonography-guided fine-needle aspiration (EUS-FNA) is a more accurate method for obtaining PNET pathological grading prior to surgery, but it is a invasive examination and greatly affected by the location and depth of tumors [12, 13]. Imaging examination plays a crucial role in diagnosing, localizing, staging, and evaluating the effectiveness of PNETs, and it aids in qualitatively and differentially diagnosing tumors [14]. Traditional imaging examinations, such as CT and MRI, can be utilized for the diagnosis of PNETs, but they offer limited information in reflecting the heterogeneity and predicting the pathological grading of tumors [15].

Radiomics, as an emerging technique, can non-invasively and quantitatively examine the imaging features contained in images, comprehensively reflect tumor heterogeneity and predict clinical or biological outcomes, and it has been widely used in the diagnosis, prognosis evaluation and curative effect monitoring of accessory diseases [16]. It sheds light on the utilization of a novel quantitative imaging approach to address the grading of PNETs.

Molecular imaging with PET/CT has become indispensable for the management of PNETs [17, 18]. It enables non-invasive tumor localization, accurate staging, tumor burden, and characterization of disease heterogeneity, while providing important prognostic information and guidance for the formulation of treatment strategies [19–21]. Somatostatin receptor 2 (SSTR 2) is highly expressed in NETs [22]. DOTATATE (DOTA,Tyr(3)-octreotate) is a somatostatin analog (SSA), which shows the highest affiffiffinity for SSTR 2 [23]. ^68^Ga-DOTATATE has the superior sensitivity and specifificity of for localizing PNETs than CT or MRI. Compared with traditional imaging modalities,, ^68^Ga- DOTATATE PET/CT can provide functional and metabolic information of lesions, better reflect the progression of diseases, have advantages in tumor heterogeneity, and provide more valuable clinical information.

Thus, we aim to build a predictive model to noninvasively achieve PNET grading. Together, we will investigated the potential clinical value of radiological variables and the rad-score based on ^68^Ga-DOTATATE PET/CT.

## Materials and methods

### Patients

The study was approved by the ethics committee of our hospital. The patients with PNETs confirmed by postoperative pathology in our hospital from January 2019 to January 2023 were included retrospective analysis. Inclusion criteria: (a) Patients with PNETs proven by postoperative pathology; (b) Clear histopathological grading; (c) ^68^Ga-DOTATATE PET/CT examination performed within 1 month before surgery; (d) Without other malignant tumors. Exclusion criteria: (a) History of receiving systemic or local anti-tumor treatment before surgery, such as radiation therapy, chemotherapy, nuclear therapy, etc; (b) Biopsy; (c) Incomplete clinical data; (d) Obvious artifacts or poor PET/CT image quality; (e) Patients with recurrent lesions.

According to the inclusion and exclusion criteria, a total of 41 individuals were included, including 21 males and 20 females, aged from 33 to 82 years, with an average age of 51.37 ± 11.09 years.

### Histopathological grading

**Fig. 1:**
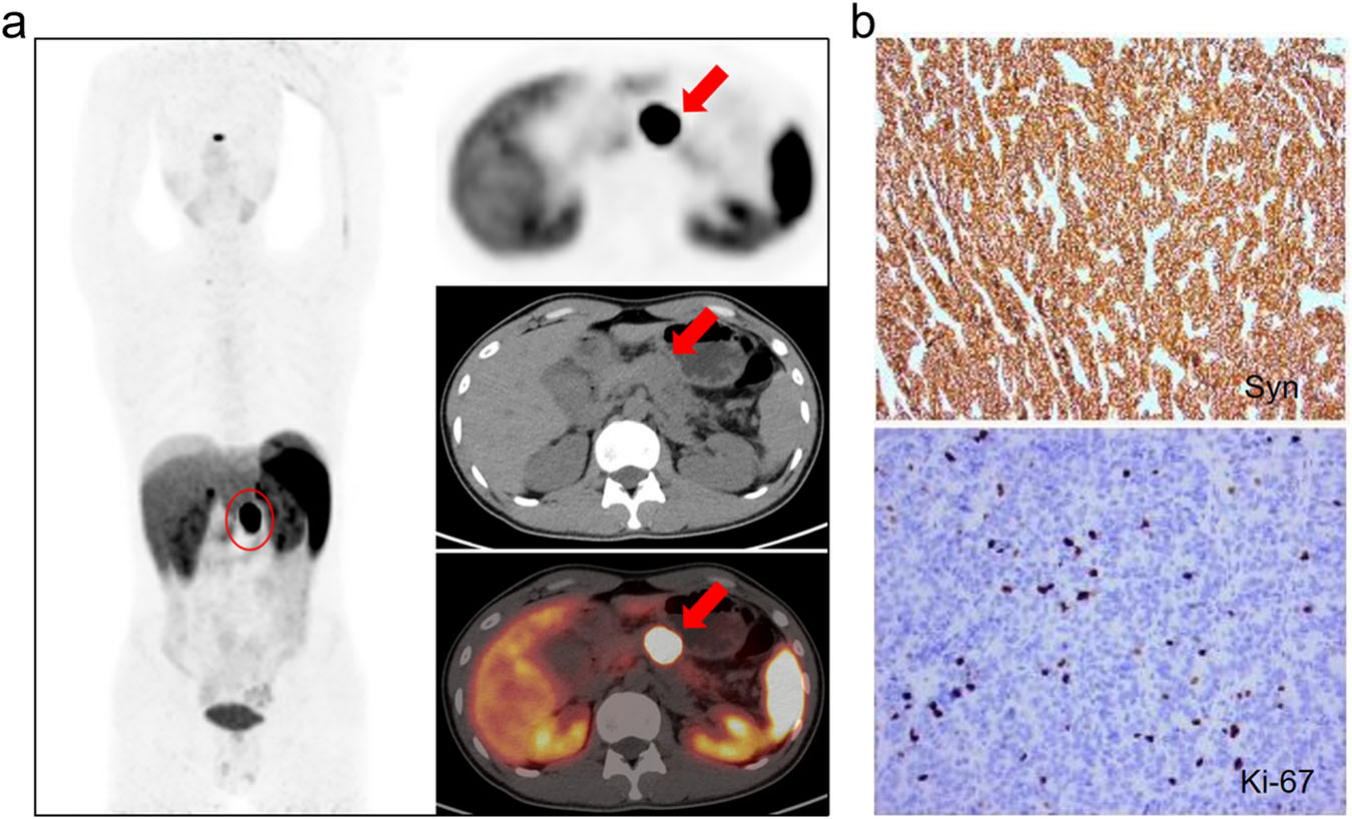
**a** Case report of pancreatic neuroendocrine tumor ^68^Ga-DOTATATE PET/CT revealed an isodense nodule with a high level of metabolic expression (SUVmax 56.6) in the pancreatic body. **b** Syn(_**+**_); Ki-67 (+, 10%)

According to the 2019 WHO guidelines for pathological classification [4], the histological grade of PNETs was confirmed. According to the number of mitoses (per 10 high-power fields, HPF) and Ki-67 index, PNETs are classified into grades 1–3, with grade 1: <2/10HPF and Ki-67 index ranging from 0 to 2%; grade 2: 3-20/10HPF, with Ki-67 index ranging from 3 to 20%; grade 3: >20/10HPF, Ki-67 index >20%. There is a typical case report shown in Fig. 1.

### Acquisition of PET/CT

All PET imaging was performed using the PET/CT scanner (uMI780, United Imaging Healthcare). Detailed configuration of this scanner can be found in previous study [24]. The resulting data were provided to a post-processing workstation (Version R002, uWS-MI, United Imaging Healthcare). The doses of ^68^Ga-DOTATATE via intravenous injection were 3.7 and 2.0 MBq/kg, respectively. The patients were instructed to drink adequate water, rest at a quiet and suitable temperature for 45–60 min after intravenous injection of imaging agent, empty the bladder, and perform CT scans from the top of the head to the upper middle of the thighs. Scanning parameters were as follows: tube voltage = 120 kV, tube current = 100 mAs, and layer thickness = 6 mm. After the completion of CT scanning, PET 3D acquisition was performed with 3 min/bed, 9–11 total beds subsequently. After the acquisition, attenuation correction was performed with CT data. PET images were reconstructed iteratively to generate cross-sectional, coronal plane, sagittal plane sectional images and 3D projected images.

### Image analysis

The PET, CT, and fused PET/CT images were analyzed by two experienced nuclear medicine physicians. When there was a difference in opinion, they concluded after discussing it in detail. The image features analysis involves qualitative and quantitative assessments [25].

Qualitative assessments: ① tumor location: pancreatic head and neck (non uncinate process area), pancreatic uncinate process, pancreatic body/tail, or multiple parts in the pancreas; ② calcification; ③ texture of pancreatic tumors: according to the proportion of cystic and solid components, they are divided into solid type, cystic solid type, and cystic type [15]; ④ pancreatic duct dilation:the widest part of the pancreatic duct with a diameter exceeding 0.35 cm is defined as pancreatic duct dilation; ⑤ bile duct dilation: Measure the widest part of the common bile duct at the upper edge of the pancreas, with a diameter greater than 0.9 cm is defined as expansion; ⑥ peripheral organ invasion: whether it affects the spleen, duodenum, and stomach; ⑦ lymph node metastasis: regional lymph node metastasis occurs; ⑧ distant metastasis: presence of liver, bone, lung, or distant metastasis. Quantitative assessments: ① size: represented by the maximum diameter line of the tumor measured in cross-section, in cm; ② metabolic parameters: the volume of interest (VOI) was selected by experienced nuclear medicine physicians, and subsequently, standardized uptake value (SUVmax), mean standardized uptake value (SUVmean), peak standardized uptake value (SUVpeak), metabolic tumor volume (MTV), and total lesion glycolysis (TLG) of primary tumor were calculated [26].

### Image segmentation and radiomic feature extraction

Each patient’s PET/CT images were imported into 3D slicer (version 5.0.2) software in DICOM format for segmentation of regions of interest (ROIs) in the lesion. After semi-automatically delineating the lesions on the axial ^68^Ga-DOTATATE PET/CT images, the ROIs were manually adjusted along the lesion margin layer by layer to 1-2 mm outside of the lesion edge to avoid partial volume effect. 107 radiomic features, including 14 shape features, 18 first-order features, 24 gray-level co-occurrence matrix features, 16 gray-level run-length matrix features, 14 gray-level dependence matrix features, 16 gray-level size zone matrix features, and 5 neighboring gray-level difference matrix features, were extracted from the two ROIs using the PyRadiomics software package in 3D slicer. These features characterize the heterogeneity of lesions and potentially reflect changes in image structure [27]. The two nuclear medicine physicians manually edited and selected enhanced features manually, aiming to guarantee the reproducibility and repeatability of radiomic features [28]. The intra- and inter-class correlation coefficients (ICCs) calculated from 15 segmented lesions in the CT images were used to determine the intra- and inter-observer reproducibility of the radiomic features. Features with the ICCs higher than 0.80 were obtained in the following analysis.

### Selection of radiomic features

We selected 28 cases as the training data set (19/9 = positive/negative). We also selected another 13 cases as the independent testing data set (9/4 = positive/negative). The training set was used to examine the robustness of radiomic features and to construct prediction models, whereas the testing set was used to validate the reliability of the prediction models. To remove the unbalance of the training data set, Synthetic Minority Oversampling TEchnique (SMOTE) was used to balance positive/negative samples. To normalize the feature matrix, we calculated the L2 norm for each feature vector and divided by it, resulting in unit vectors. Since the feature space was high-dimensional, we compared the similarity of each feature pair using Pearson correlation coefficient (PCC) and removed one feature from pairs with PCC values larger than 0.99. This reduced the dimension of the feature space and ensured that each feature was independent. Before building the model, we used recursive feature elimination (RFE) to select the most important features based on a classifier by considering smaller subsets of the features recursively. For classification, we utilized logistic regression which is a linear classifier and combines multiple features. In a high-dimensional space, a hyper-plane is used to separate the samples. To determine the hyper-parameter (e.g. the number of features) of model, we applied 5-fold cross-validation on the training data set. The performance of the model on the validation set was used to determine the optimal hyper-parameters. The performance of the radiomic feature model was evaluated using receiver operating characteristic (ROC) curve analysis and the area under the curve (AUC) was calculated for quantification. The accuracy, sensitivity, specificity, positive predictive value (PPV), and negative predictive value (NPV) were also calculated at a cutoff value that maximized the value of the Yorden index. We also estimated the 95% confidence interval by bootstrape with 1000 samples. All above processes were implemented with FeAture Explorer Pro (FAEPro, V 0.4.2) on Python (3.7.6).

### Development, evaluation and comparison of model

Univariable analysis was utilized to assess the association between PNET grading and clinicopathological/radiological factors. The variance inflation factor (VIF) was used to assess the collinearity of each variable, and variables with a VIF less than 10 were obtained in the following analyses. Potential clinical risk factors (*P* < 0.05) related to the histologic grade were included for clinical model building with logistic regression. The radiomic score(rad-score) was calculated for each patient to show the prediction risk of grade 2/3 via the radiomic signature. The relationship between rad-score and histologic grade was explored by using a t-test. Subsequently, a comprehensive model combining rad-score and clinical features was established. The ROC curve were plotted and AUCs were utilized to quantify the discriminative ability of each model. The delong test was used to compare the performance of two prediction models.

### Development and validation of the nomogram

A nomogram was developed based on the proposed comprehensive model as a graphical presentation. The nomogram is a simple and user-friendly clinical tool, which can visually display the predicted outcome for each patient. The Hosmer–Lemeshow test was used to evaluate the goodness-of-fit of the model. In addition, the decision curve analysis (DCA) was conducted to validate the clinical utility of the nomogram by estimating the net benefit at various threshold probabilities, synthesizing true-positive and false-positive rates.

### Statistical analysis

SPSS Statistics (version 21.0) was used for statistical analysis. Quantitative variables were assessed for differences between the grade 1 and grade 2/3 groups using the t-test or Mann–Whitney U test, while qualitative variables were analyzed using the chi-square test or Fisher’s exact test. *P* < 0.05 was deemed statistically significant.

## Result

### Clinical characteristics

A total of 41 patients with PNETs in this study were diagnosed through surgical resection and pathological biopsy. Among them, 4 patients underwent pancreaticoduodenectomy, 18 patients underwent distal pancreatectomy, and 19 patients underwent total pancreatectomy. 14 patients (34.1%) were categorized as grade1, 18 patients (44.0%) were grade 2 and 9 patients (21.9%) were grade 3. 9 patients had invasion of surrounding organs, including 4 cases of spleen invasion, 4 cases of descending duodenum invasion, and 1 case of adrenal gland invasion; 8 patients had local lymph node metastasis. A total of 26 patients had distant metastasis, of whom 6 had only intrahepatic metastasis, 5 had metastasis to other organs excluding the liver, and 15 had metastases in the bone, lungs, and other organs including the liver. There were no differences between the two groups in terms of age, gender, and BMI. (*p* > 0.05).

### Radiological characteristics

Qualitative assessments: There were significant statistical differences were observed in adjacent organ invasion, N staging, and M staging between grade 1 and grade 2/3 (*p* < 0.05). There were no statistically significant differences found in tumor location, the presence or absence of pancreatic duct dilation, calcification, and tumor texture (*p* > 0.05). Quantitative assessments: There were no statistically significant differences observed in terms of maximum tumor diameter, SUVmax, SUVmean, SUVpeak, MTV, and TLG of the primary tumor (*p* > 0.05). They were shown in Table 1.

**Table 1:** clinical and radiological characteristics

Total (*n* = 41)	grade 1 (*n* = 14)	Grade 2/3 (*n* = 27)	*P*
Age, mean ± SD	49.2 ± 13.6	52.5 ± 9.6	0.317
Sex(%)			0.91
Male	7(50%)	14(52%)	
Female	7(50%)	13(48%)	
BMI			0.532
thin	0(0%)	3(11%)	
Standard	9(64%)	15(56%)	
Overweight	3(22%)	7(26%)	
Obesity	2(14%)	2(7%)	
Maximum diameter (x ±s,cm)	3.9 ± 1.8	4.2 ± 3.0	0.111
Location			0.686
Pancreatic head and neck	5(36%)	7(26%)	
Pancreatic body and tail	8(57%)	16(59%)	
Uncinate process area	1(7%)	4(15%)	
pancreatic duct			0.733
Dilation	12(86%)	7(26%)	
No dilation	2(14%)	20(74%)	
Calcification			0.75
Yes	3(22%)	7(26%)	
No	11(78%)	20(74%)	
Composition			0.574
Solidity	11(78%)	19(70%)	
Cystic solidity	3(22%)	8(30%)	
Peripheral organ invasion			0.0142
Yes	0(0%)	9(33%)	
No	14(100%)	18(67%)	
Local lymph node metastasis			0.035
Yes	0(0%)	8(30%)	
No	14(100%)	19(70%)	
Distant metastasis			0.041
No	9(65%)	6(22%)	
Only liver	1(7%)	5(19%)	
Other organs	2(14%)	3(11%)	
Both liver and other organs	2(14%)	13(48%)	
SUVmax	30.80 ± 19.77	23.34 ± 20.46	0.249
SUVmean	19.15 ± 13.6	14.27 ± 13.10	0.330
SUVpeak	23.28 ± 15.37	17.72 ± 14.00	0.382
MTV	21.32 ± 42.73	29.54 ± 50.49	0.204
TLG	173.75 ± 235.19	237.61 ± 414.12	0.726

### Selection of radiomic features

**Fig. 2:**
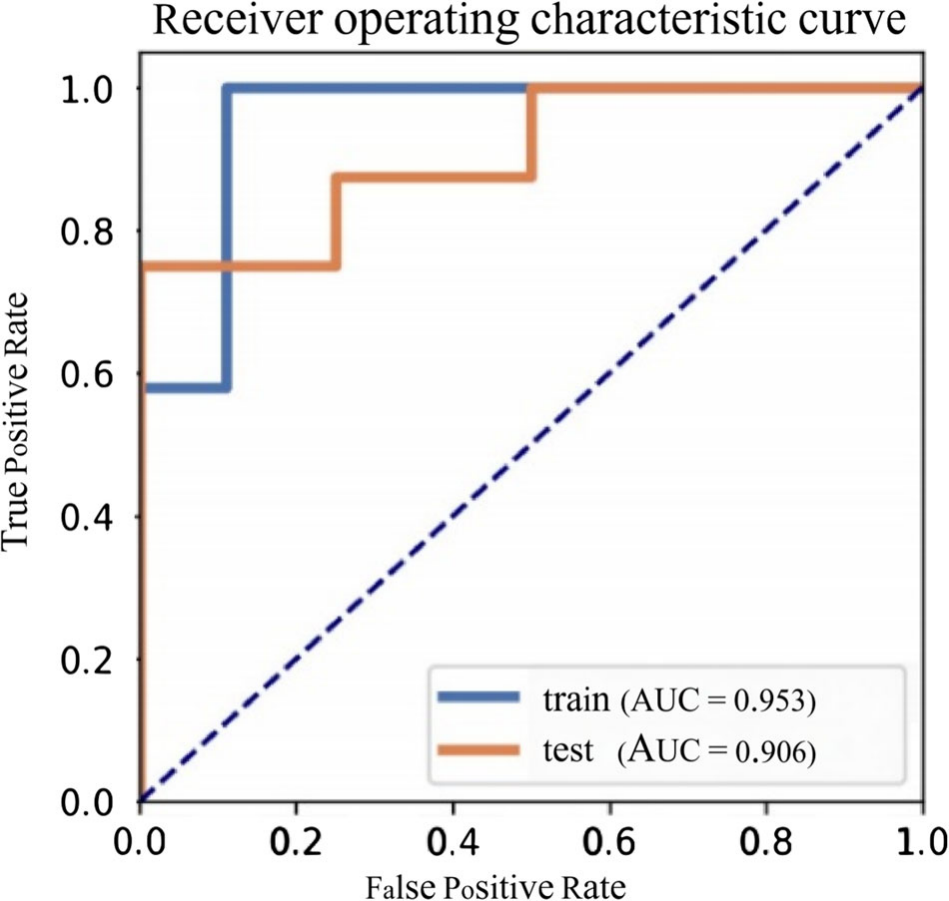
ROC curves of radiomic feature model in the train and test cohorts

Through dimensionality reduction, 12 optimal features were selected, including 3 PET radiomic features and 9 CT radiomic features. These features and corresponding coefficients are shown in Table 2. We found that the model based on 12 features can get the highest AUC on the validation data set. The AUC and the accuracy could achieve 0.409 and 0.429, respectively. In this point, The AUC and the accuracy of the model achieve 0.906 and 0.833 on testing data set. The ROC curve was shown in Fig. 2.

**Table 2:** The coefficients of features in the model

Features	Coef in model
original_shape_Maximum2DDiameterColumn	7.505
original_firstorder_Entropy	3.036
original_firstorder_InterquartileRange	−2.295
origina _firstorder_MeanAbsoluteDeviation	0.329
original_glcm_Contrast	1.168
original_glcm_MCC	−2.273
original_glrlm_RunLengthNonUniformity	−6.354
original_glrlm_RunVariance	5.207
original_glszm_SmallAreaLowGrayLevelEmphasis	2.751
pet_original_firstorder_Kurtosis	−2.225
pet_original_firstorder_Skewness	−4.229
pet_original_gldm_LargeDependenceHighGrayLevelEmphasis	5.428

### Development, evaluation and comparison of model

**Fig. 3:**
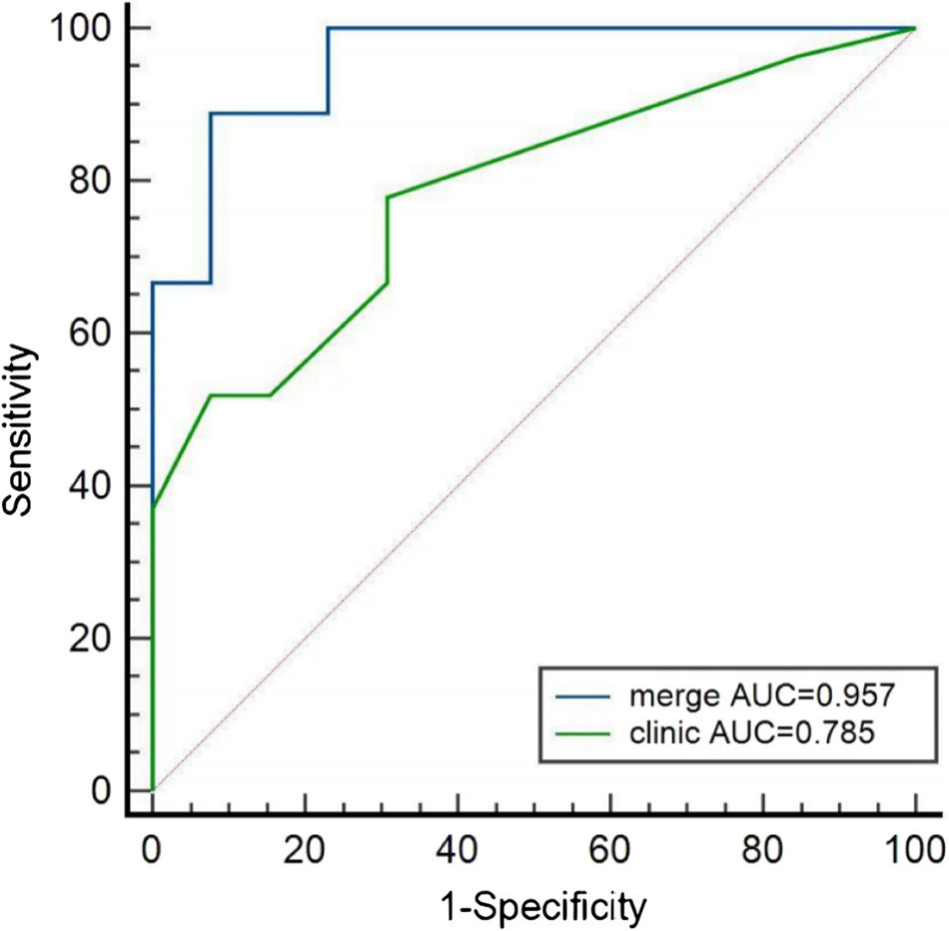
Comparison of ROC curves of the clinical model and the comprehensive model merging clinical risk factors and rad-score

Three clinical variables (adjuvant organic invasion, N staging, and M staging) were used to construct a clinical model with AUC of 0.785. There were significant differences in rad-score between grade 1 and grade 2/3 (*p* < 0.05). When combining clinical variables and rad-score, the comprehensive model displayed the best predictive performance with AUC of 0.957. The ROC curve analysis of the two models was shown in Fig. 3. The effectiveness of the comprehensive model in predicting PNET grading has been further improved, significantly higher than clinical models (delong test, *P* = 0.0142).

### Development and validation of the nomogram

**Fig. 4:**
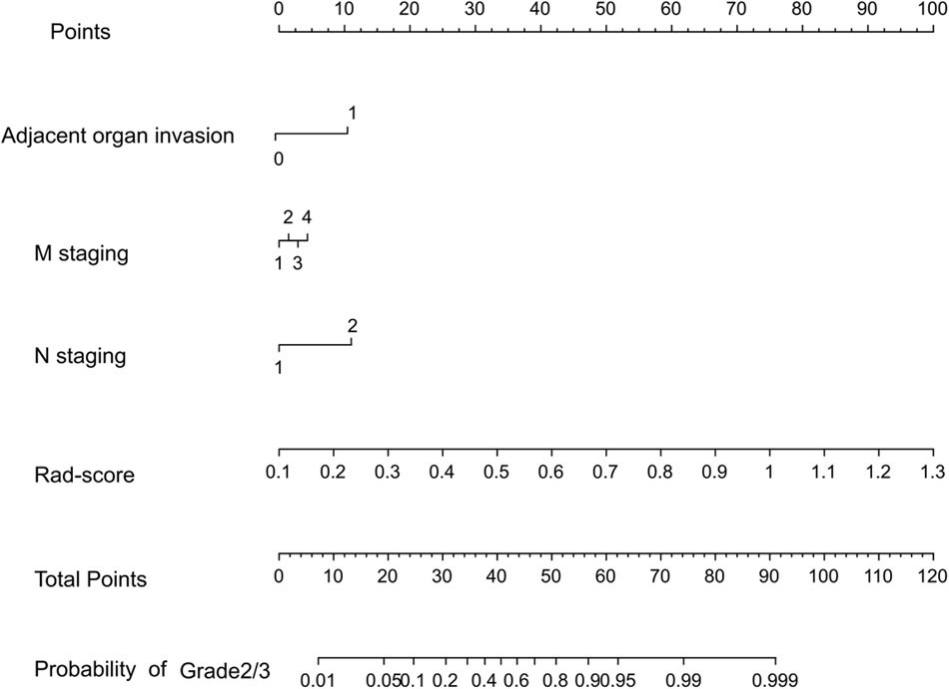
The nomogram based on the comprehensive model incorporating the clinical risk factors and the rad-score

Considering that the comprehensive model has good predictive ability, a nomogram based on the comprehensive model which can provide the probability of grade 2/3 for PNETs is established. The nomogram in Fig. 4 displayed the advantages of personalized prediction. By considering individuals’ clinical information and rad-score value, one can determine the margin and draw a vertical line towards the points axis to find the corresponding score. After summing up the scores for each point, one can locate the total on the total points axis to estimate the probability of grade 2/3.

**Fig. 5:**
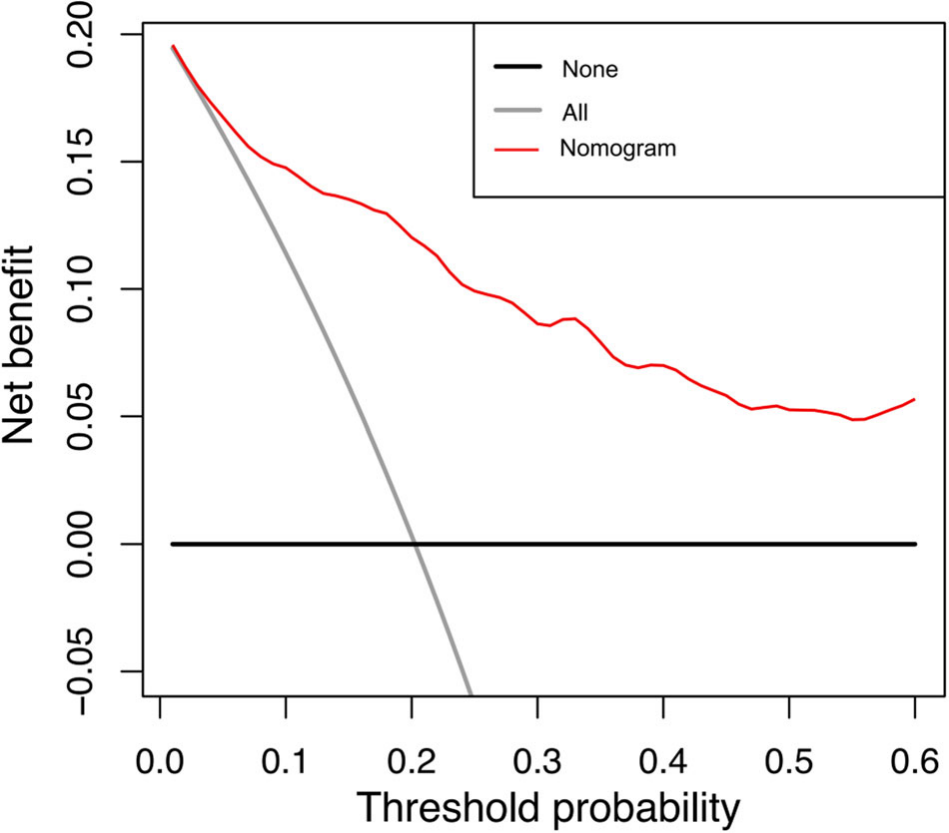
Decision curves for the comprehensive model

The nomogram model was shown to have excellent goodness-of-fit through the Hosmer–Lemeshow test. Figure 5 presented the DCA for the comprehensive model, where the red line indicated the benefit of the comprehensive model, the gray line reflected the assumption of treating all patients as grade 2/3 (“treat all”), and the black line represented the assumption of not treating any patients as grade 2/3 (“treat none”). The comprehensive model displayed the optimal net benefit.

## Discussion

In this study, we proposed an optimal model integrating clinical risk factors and rad-score from 68Ga-DOTATATE PET/CT images for individually predicting histologic grade of PNETs. Firstly, the radiomic analysis showed the rad-score was significantly associated with PNET grading, which was in line with previous studies [29, 30]. Previous study also highlighted the limited accuracy of conventional image anlaysis for PET and radiological imaging and therefore the need for more sophisticated anlaysis such as radiomics [31]. These indicated that the mathematical objectivity of radiomics allows for accurate evaluation of histologic grade of PNETs using ^68^Ga-DOTATATE PET/CT, instead of other invasive methods. Secondly, the statistical analysis indicated that the adjacent organ invasion, N staging, and M staging were the clinical factors mostly related to the grade. However, the clinical model building with it had limited performance. Therefore, we developed the comprehensive model to test whether the rad-score and clinical factor were complementary. Atkinson, Charlotte et al’s study also suggested that radiomics analysis of NETs based on ^68^Ga-DOTATATE PET/CT might have prognostic significance and represent a useful complement to the evaluation of patients [32]. The comprehensive model combining clinical variables and rad-score obtained the most ideal performance; thereby, we considered it as a powerful tool for the prediction of PNET grading and clinical decisionmaking.

Previous studies have investigated the relationship between PNET grading and the radiomics analysis based on enhanced CT images [29, 30, 33, 34]. But the acquisition of the radiomic signature was based on arterial and/or portal venous phase CT images, which may be affected by the scan time, scan parameters, and the use of contrast agents [35]. These factors can potentially impact the generalizability of predictive model and may be limited for individuals who are contraindicated for contrast agents. In addition, traditional CT images may provide limited information in fully characterizing lesions, whereas PET imaging parameters can provide complementary functional and metabolic information at the molecular level [36]. Some recent previous studies have indicated that the potential role of radiomics analysis derived from preoperative PET/CT or PET/MRI in the noninvasively predicting specific tumor characteristics and outcome of patients with PNENs [37–39]. So the combination of CT images and PET imaging can provide more comprehensive understanding of the pathology.

The adjacent organ invasion, N staging, and M staging were indicated as the clinical factors mostly related to the grade. These clinical factors were mostly existed in grade 2/3 of PNETs. Namely, the tumors in PNET patients with higher grade tend to show more infiltration into the surrounding tissue, higer N stage, and higher M stage than lower-grade tumors, which was consistently demonstrated in previous studies [40, 41]. This might be the reason why advanced histologic grade commonly indicate a poor prognosis of PNETs [42, 43].

For the radiomic features selection, we have identified 12 radiomic features that can be used to characterize PNETs. Various pathological tumor types have different values of radiomic features, which could elucidate the underlying mechanism for utilizing radiomics in tumor classification [44]. So the selected radiomic features to some extent reflect the morphological, texture, and other characteristics exhibited by tumors, and can be used to assist physicians in disease diagnosis and pathological grading.

To explore clinical applications, we proposed and validated a nomogram model integrating clinical risk factors and rad-score as a potent tool to predict PNET grading and help clinical decisions. We found that in the nomogram, the rad-score had significantly higher weight for grade of PNETs compared to other clinical variables. This finding can be explained by the fact that radiomics analysis extracts 3D imaging information of the tumor, providing a comprehensive and objective characterization of the tumors compared to traditional lesion assessment methods [16, 45]. In addition, the comprehensive model has significantly higher predictive performance than the clinical model. This suggests that the combination of radiomic features and clinical factors can be fully exploited and more effective in predicting disease. Moreover, there was a correlation between radiological features and clinical features such as tumor prognosis and staging [46]. Therefore, combining radiomic and clinical features can increase the information crossover between data sets, which can improve the accuracy and robustness of the predictive model [30, 47].

This study has several limitations. Firstly, it was a small retrospective, single-center investigation, and the model should be externally validated with a larger patient sample size. Secondly, only a few clinical features were included, and there were confounding variables such as the M staging, which might be correlated with radiomic features extracted from the primary tumor lesion. Thirdly, while the radiomic model based on logistic regression demonstrated good predictive performance, future research is needed to apply various machine learning algorithms to establish prediction models and find the optimal modeling approach.

## Conclusion

The radiomic features of 68Ga-DOTATATE PET/CT allow for accurate and noninvasive evaluation of histologic grade of PNETs. And we successfully proposed a a comprehensive nomogram model with powerful predictive capability for grade 1 and grade 2/3 of PNETs, which could assist in the clinical diagnosis and decision-making of patients with PNETs.

## Abbreviations:

PNET: Pancreatic neuroendocrine tumor
NET: Neuroendocrine tumor
EUS-FNA: Endoscopic ultrasonography-guided fine-needle aspiration
DOTA,Tyr(3)-octreotate: DOTATATE
SSTR 2: Somatostatin receptor 2
VOI: Volume of interest
SUVmax: Maximum standardized uptake value
SUVmean: Mean standardized uptake value
SUVpeak: Peak standardized uptake value
MTV: Metabolic tumor volume
TLG: Total lesion glycolysis
ICC: Inter-class correlation coefficient
PCC: Pearson correlation coefficient
RFE: Recursive feature elimination
ROC: Receiver operating characteristic
AUC: Area under the curve
DCA: Decision curve analysis
PPV: Positive predictive value
NPV: Negative predictive value
